# Dimethyl (1-hydr­oxy-1,2-diphenyl­ethyl)phospho­nate

**DOI:** 10.1107/S1600536809004036

**Published:** 2009-02-06

**Authors:** Nurcan Acar, M. Nawaz Tahir, Hamza Yılmaz, Muhammad Saeed Ahmad Chishti, Muhammad Ali Malik

**Affiliations:** a Department of Chemistry, Faculty of Science, University of Ankara, Ankara, Turkey; b Department of Physics, University of Sargodha, Sargodha, Pakistan

## Abstract

In the mol­ecule of the title compound, C_16_H_19_O_4_P, the coordination around the P atom is distorted tetra­hedral. The aromatic rings are oriented at a dihedral angle of 72.28 (11)°. Intra­molecular C—H⋯O hydrogen bonding result in the formation of five- and six-membered rings. In the crystal structure, inter­molecular C—H⋯O hydrogen bonds link the mol­ecules. There is also a weak C—H⋯π inter­action.

## Related literature

For related structures, see: Hudson *et al.* (1993 ▶); Tahir *et al.* (2007 ▶); Wroblewski *et al.* (2000 ▶).
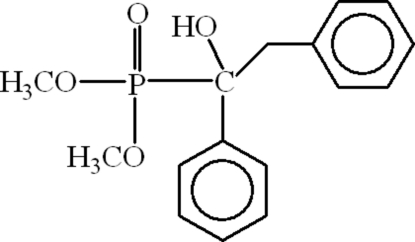


## Experimental

### 

#### Crystal data

C_16_H_19_O_4_P
*M* *_r_* = 306.28
Monoclinic, 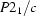

*a* = 6.0671 (12) Å
*b* = 17.0962 (11) Å
*c* = 15.0502 (12) Å
β = 95.0021 (11)°
*V* = 1555.1 (3) Å^3^
*Z* = 4
Mo *K*α radiation
μ = 0.19 mm^−1^
*T* = 296 K
0.28 × 0.12 × 0.10 mm

#### Data collection

Enraf–Nonius CAD-4 diffractometer
Absorption correction: ψ scan (North *et al.*, 1968 ▶) *T*_min_ = 0.968, *T*_max_ = 0.982
2800 measured reflections
2794 independent reflections
1655 reflections with *I* > 2σ(*I*)
*R*_int_ = 0.059
3 standard reflections frequency: 120 min intensity decay: −1.3%

#### Refinement

*R*[*F* ^2^ > 2σ(*F* ^2^)] = 0.064
*wR*(*F* ^2^) = 0.159
*S* = 1.01
2794 reflections
191 parameters
H-atom parameters constrained
Δρ_max_ = 0.40 e Å^−3^
Δρ_min_ = −0.35 e Å^−3^

### 

Data collection: *CAD-4 EXPRESS* (Enraf–Nonius, 1994 ▶); cell refinement: *CAD-4 EXPRESS*; data reduction: *XCAD4* (Harms & Wocadlo, 1995 ▶); program(s) used to solve structure: *SHELXS86* (Sheldrick, 2008 ▶); program(s) used to refine structure: *SHELXL97* (Sheldrick, 2008 ▶); molecular graphics: *ORTEP-3 for Windows* (Farrugia, 1997 ▶) and *PLATON* (Spek, 2003 ▶); software used to prepare material for publication: *WinGX* (Farrugia, 1999 ▶).

## Supplementary Material

Crystal structure: contains datablocks global, I. DOI: 10.1107/S1600536809004036/hk2619sup1.cif

Structure factors: contains datablocks I. DOI: 10.1107/S1600536809004036/hk2619Isup2.hkl

Additional supplementary materials:  crystallographic information; 3D view; checkCIF report

## Figures and Tables

**Table 1:** Hydrogen-bond geometry (Å, °)

*D*—H⋯*A*	*D*—H	H⋯*A*	*D*⋯*A*	*D*—H⋯*A*
O1—H1⋯O2^i^	0.8200	1.9000	2.710 (3)	172.00
C2—H2⋯O4	0.9300	2.5200	2.956 (4)	109.00
C6—H6⋯O1	0.9300	2.3000	2.671 (4)	103.00
C14—H14⋯O1^ii^	0.9300	2.5700	3.432 (5)	154.00
C16—H16*A*⋯O3^ii^	0.9600	2.4700	3.336 (5)	149.00
C15—H15*B*⋯CgA	0.9600	2.7200	3.608 (5)	154.00

Symmetry codes: (i) 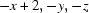; (ii) 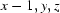. CgA is centroid of the C1–C6 ring.

**Fig. 1.**
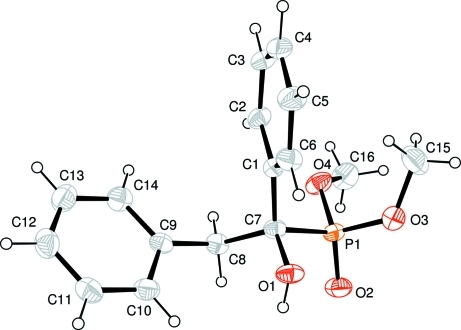
The molecular structure of the title molecule, with the atom-numbering scheme. Displacement ellipsoids are drawn at the 50% probability level.

**Fig. 2.**
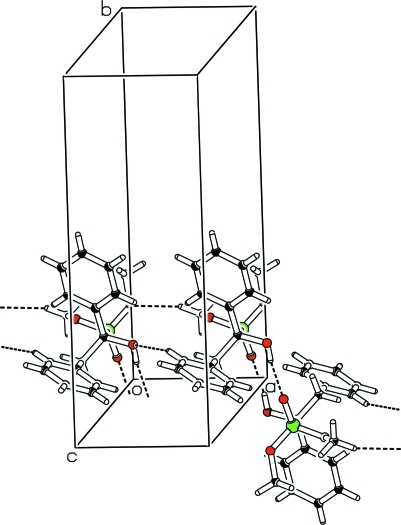
A partial packing diagram of the title compound. Hydrogen bonds are shown as dashed lines.

## supplementary crystallographic information

### Comment

The crystal structure of (*R*)-dimethyl [(2-chlorophenyl)hydroxymethyl]- phosphonate (Tahir *et al.*, 2007), which is a member of α-hydroxy phosphonates, has been reported, previously. In continuation to the study of such organic compounds, we report herein the synthesis and crystal structure of the title compound, (I).

In the molecule of (I), (Fig. 1) the coordination around the P atom is a distorted tetrahedral. The crystal structure of dimethyl α-chloromethyl-α -hydroxybenzylphosphonate, (II) (Hudson *et al.*, 1993) and C_24_H_26_NO_4_P, (III) (Wroblewski *et al.*, 2000) have also been reported, in which both of them have similar coordinations around the C-atom having α-hydroxy group. In (I), the benzene rings A (C1-C6) and B (C9-C14) are oriented at a dihedral angle of 72.28 (11)°. The intramolecular C-H···O hydrogen bonds (Table 1) result in the formations of five- and six-membered rings C (O1/C1/C6/C7/H6) and D (P1/O4/C1/C2/C7/H2), respectively, having planar and boat conformations.

In the crystal structure, intermolecular O-H···O and C-H···O hydrogen bonds (Table 1) link the molecules (Fig. 2), in which they may be effective in the stabilization of the structure. There is also a weak C—H···π interaction (Table 1).

### Experimental

For the preparation of the title compound, 2-phenylacetophenone (1.96 g, 10 mmol) was disolved in dimethylphosphonate (1.10 g, 10 mmol)  at room temperature. Then, KF (2.5 g) and ψ-Al_2_O_3_ (2.5 g) were added partwise and the mixture was kept at room temperature for 72 h. The product was extracted twice with 50 ml portions of a dichloromethane/methanol mixture (1:1). After the evaporation of the solvent on a rotary evaporator, the residue was crystallized from a mixture of diethyl ether/acetone (3:1) (m.p. 414 K).

### Refinement

H atoms were positioned geometrically, with O-H = 0.82 Å (for OH), C-H = 0.93, 0.96 and 0.97 Å for aromatic, methyl and methylene H, and constrained to ride on their parent atoms with U_iso_(H) = xU_eq_(C), where x = 1.5 for methyl H, and x = 1.2 for all other H atoms.

### Crystal data

**Table:**

C_16_H_19_O_4_P	*F*(000) = 648
*M**_r_* = 306.28	*D*_x_ = 1.308 Mg m^−^^3^
Monoclinic, *P*2_1_/*c*	Melting point = 83–86 K
Hall symbol: -P 2ybc	Mo *K*α radiation, λ = 0.71073 Å
*a* = 6.0671 (12) Å	Cell parameters from 15 reflections
*b* = 17.0962 (11) Å	θ = 10.0–11.3°
*c* = 15.0502 (12) Å	µ = 0.19 mm^−^^1^
β = 95.0021 (11)°	*T* = 296 K
*V* = 1555.1 (3) Å^3^	Needle, colorless
*Z* = 4	0.28 × 0.12 × 0.10 mm

### Data collection

**Table:**

Enraf–Nonius CAD-4 diffractometer	*R*_int_ = 0.059
ω/2θ scans	θ_max_ = 25.2°, θ_min_ = 2.4°
Absorption correction: ψ scan (North *et al.*, 1968)	*h* = 0→7
*T*_min_ = 0.968, *T*_max_ = 0.982	*k* = 0→20
2800 measured reflections	*l* = −18→17
2794 independent reflections	3 standard reflections every 120 min
1655 reflections with *I* > 2σ(*I*)	intensity decay: −1.3%

### Refinement

**Table:**

Refinement on *F*^2^	Primary atom site location: structure-invariant direct methods
Least-squares matrix: full	Secondary atom site location: difference Fourier map
*R*[*F*^2^ > 2σ(*F*^2^)] = 0.064	H-atom parameters constrained
*wR*(*F*^2^) = 0.159	*w* = 1/[σ^2^(*F*_o_^2^) + (0.088*P*)^2^] where *P* = (*F*_o_^2^ + 2*F*_c_^2^)/3
*S* = 1.01	(Δ/σ)_max_ < 0.001
2794 reflections	Δρ_max_ = 0.40 e Å^−^^3^
191 parameters	Δρ_min_ = −0.35 e Å^−^^3^
0 restraints	

### Special details

**Table:**

Geometry. Bond distances, angles *etc*. have been calculated using the rounded fractional coordinates. All su's are estimated from the variances of the (full) variance-covariance matrix. The cell e.s.d.'s are taken into account in the estimation of distances, angles and torsion angles

### Fractional atomic coordinates and isotropic or equivalent isotropic displacement parameters (Å^2^)

**Table:**

	*x*	*y*	*z*	*U*_iso_*/*U*_eq_	
P1	0.79483 (14)	0.12098 (5)	−0.05193 (6)	0.0416 (3)	
O1	1.0479 (3)	0.10996 (12)	0.09544 (16)	0.0460 (8)	
O2	0.8463 (4)	0.04357 (13)	−0.08487 (15)	0.0576 (9)	
O3	0.9546 (5)	0.18125 (15)	−0.08944 (18)	0.0659 (10)	
O4	0.5517 (4)	0.14911 (15)	−0.07722 (15)	0.0582 (9)	
C1	0.7793 (5)	0.21108 (17)	0.1007 (2)	0.0368 (10)	
C2	0.5716 (5)	0.24481 (19)	0.0869 (2)	0.0444 (11)	
C3	0.5409 (6)	0.3227 (2)	0.1076 (2)	0.0533 (12)	
C4	0.7133 (7)	0.3671 (2)	0.1449 (3)	0.0567 (14)	
C5	0.9175 (7)	0.3333 (2)	0.1623 (3)	0.0555 (14)	
C6	0.9516 (6)	0.25648 (18)	0.1401 (2)	0.0470 (11)	
C7	0.8226 (5)	0.12778 (17)	0.0709 (2)	0.0387 (10)	
C8	0.6654 (5)	0.06725 (18)	0.1090 (2)	0.0435 (11)	
C9	0.6703 (6)	0.06607 (17)	0.2091 (2)	0.0411 (10)	
C10	0.8471 (6)	0.03455 (19)	0.2618 (2)	0.0522 (12)	
C11	0.8457 (7)	0.0313 (2)	0.3538 (2)	0.0573 (14)	
C12	0.6657 (8)	0.0583 (2)	0.3943 (3)	0.0628 (14)	
C13	0.4908 (8)	0.0895 (2)	0.3432 (3)	0.0635 (16)	
C14	0.4917 (6)	0.0931 (2)	0.2520 (3)	0.0537 (12)	
C15	0.9185 (9)	0.2641 (2)	−0.0979 (3)	0.0863 (19)	
C16	0.4445 (7)	0.1432 (3)	−0.1647 (3)	0.0733 (16)	
H1	1.06744	0.06272	0.09092	0.0552*	
H2	0.45212	0.21495	0.06361	0.0534*	
H3	0.40178	0.34518	0.09602	0.0636*	
H4	0.69192	0.41953	0.15824	0.0679*	
H5	1.03397	0.36254	0.18933	0.0666*	
H6	1.09151	0.23458	0.15143	0.0565*	
H8A	0.51529	0.07819	0.08461	0.0520*	
H8B	0.70402	0.01557	0.08879	0.0520*	
H10	0.96854	0.01529	0.23501	0.0623*	
H11	0.96685	0.01083	0.38820	0.0688*	
H12	0.66330	0.05520	0.45591	0.0750*	
H13	0.36975	0.10860	0.37031	0.0764*	
H14	0.37005	0.11401	0.21825	0.0644*	
H15A	1.04258	0.28789	−0.12280	0.1294*	
H15B	0.90211	0.28613	−0.04020	0.1294*	
H15C	0.78662	0.27362	−0.13644	0.1294*	
H16A	0.29675	0.16336	−0.16523	0.1095*	
H16B	0.43893	0.08928	−0.18277	0.1095*	
H16C	0.52532	0.17277	−0.20525	0.1095*	

### Atomic displacement parameters (Å^2^)

**Table:**

	*U*^11^	*U*^22^	*U*^33^	*U*^12^	*U*^13^	*U*^23^
P1	0.0398 (5)	0.0398 (5)	0.0458 (5)	0.0101 (4)	0.0066 (4)	−0.0001 (4)
O1	0.0323 (13)	0.0371 (12)	0.0669 (15)	0.0059 (10)	−0.0053 (11)	−0.0040 (11)
O2	0.0693 (17)	0.0495 (15)	0.0533 (14)	0.0195 (13)	0.0007 (12)	−0.0048 (11)
O3	0.0681 (18)	0.0571 (17)	0.0766 (17)	0.0082 (13)	0.0295 (14)	0.0097 (13)
O4	0.0503 (15)	0.0781 (17)	0.0450 (14)	0.0233 (13)	−0.0034 (11)	−0.0082 (12)
C1	0.0363 (18)	0.0357 (17)	0.0389 (17)	0.0016 (14)	0.0064 (14)	0.0009 (13)
C2	0.0339 (19)	0.047 (2)	0.053 (2)	0.0006 (15)	0.0075 (14)	−0.0066 (15)
C3	0.051 (2)	0.051 (2)	0.060 (2)	0.0171 (19)	0.0163 (18)	−0.0002 (17)
C4	0.071 (3)	0.0351 (19)	0.067 (2)	0.0015 (19)	0.024 (2)	−0.0090 (17)
C5	0.057 (3)	0.041 (2)	0.069 (2)	−0.0050 (18)	0.0085 (19)	−0.0118 (17)
C6	0.0391 (19)	0.042 (2)	0.059 (2)	−0.0002 (15)	0.0000 (16)	0.0002 (16)
C7	0.0306 (18)	0.0371 (17)	0.0476 (18)	0.0025 (14)	−0.0013 (14)	−0.0015 (14)
C8	0.0370 (19)	0.0395 (18)	0.053 (2)	−0.0038 (14)	−0.0009 (15)	0.0002 (15)
C9	0.0426 (19)	0.0294 (16)	0.0501 (19)	−0.0046 (14)	−0.0022 (16)	−0.0009 (14)
C10	0.054 (2)	0.048 (2)	0.054 (2)	0.0105 (17)	0.0010 (17)	0.0012 (16)
C11	0.063 (3)	0.053 (2)	0.054 (2)	0.0051 (19)	−0.0051 (19)	0.0080 (18)
C12	0.085 (3)	0.059 (2)	0.045 (2)	−0.010 (2)	0.009 (2)	−0.0025 (18)
C13	0.062 (3)	0.068 (2)	0.063 (3)	0.000 (2)	0.019 (2)	−0.004 (2)
C14	0.041 (2)	0.055 (2)	0.065 (2)	−0.0028 (16)	0.0034 (18)	0.0010 (17)
C15	0.131 (4)	0.050 (3)	0.082 (3)	−0.005 (3)	0.032 (3)	0.010 (2)
C16	0.061 (3)	0.098 (3)	0.059 (2)	0.012 (2)	−0.006 (2)	−0.001 (2)

### Geometric parameters (Å, °)

**Table:**

P1—O2	1.457 (2)	C12—C13	1.364 (6)
P1—O3	1.554 (3)	C13—C14	1.374 (6)
P1—O4	1.567 (3)	C2—H2	0.9300
P1—C7	1.845 (3)	C3—H3	0.9300
O1—C7	1.418 (4)	C4—H4	0.9300
O3—C15	1.437 (4)	C5—H5	0.9300
O4—C16	1.420 (5)	C6—H6	0.9300
O1—H1	0.8200	C8—H8A	0.9700
C1—C6	1.392 (5)	C8—H8B	0.9700
C1—C7	1.523 (4)	C10—H10	0.9300
C1—C2	1.385 (4)	C11—H11	0.9300
C2—C3	1.384 (5)	C12—H12	0.9300
C3—C4	1.372 (5)	C13—H13	0.9300
C4—C5	1.371 (6)	C14—H14	0.9300
C5—C6	1.375 (5)	C15—H15A	0.9600
C7—C8	1.551 (4)	C15—H15B	0.9600
C8—C9	1.504 (4)	C15—H15C	0.9600
C9—C14	1.388 (5)	C16—H16A	0.9600
C9—C10	1.386 (5)	C16—H16B	0.9600
C10—C11	1.387 (4)	C16—H16C	0.9600
C11—C12	1.376 (6)		
			
O1···O2	3.094 (3)	C9···H16B^vi^	2.7600
O1···O3	3.045 (4)	C10···H16B^vi^	2.9200
O1···C10	3.154 (4)	C10···H1	3.0400
O1···O2^i^	2.710 (3)	C11···H5^vii^	3.0600
O2···O1^i^	2.710 (3)	C12···H15C^iii^	3.0100
O2···O1	3.094 (3)	C13···H15C^iii^	2.9500
O3···C16^ii^	3.336 (5)	C16···H15C	3.0500
O3···O1	3.045 (4)	H1···O2	2.8800
O4···C2	2.956 (4)	H1···C10	3.0400
O1···H14^ii^	2.5700	H1···H8B	2.3500
O1···H6	2.3000	H1···H10	2.4400
O1···H10	2.7300	H1···O2^i^	1.9000
O1···H8A^ii^	2.9100	H2···O4	2.5200
O2···H16B	2.8700	H2···C8	2.8900
O2···H8B^i^	2.9100	H2···H8A	2.3900
O2···H10^i^	2.8000	H5···C11^viii^	3.0600
O2···H1^i^	1.9000	H6···O1	2.3000
O2···H8B	2.8600	H8A···O1^v^	2.9100
O2···H1	2.8800	H8A···O4	2.7500
O3···H16A^ii^	2.4700	H8A···C2	2.8700
O4···H2	2.5200	H8A···H2	2.3900
O4···H8A	2.7500	H8A···H14	2.3500
O4···H15C	2.7500	H8B···O2	2.8600
C1···C15	3.303 (5)	H8B···H1	2.3500
C2···O4	2.956 (4)	H8B···O2^i^	2.9100
C2···C9	3.590 (4)	H8B···H16B^vi^	2.4900
C3···C16^iii^	3.574 (5)	H10···O1	2.7300
C4···C16^iii^	3.423 (6)	H10···H1	2.4400
C6···C15	3.572 (5)	H10···O2^i^	2.8000
C9···C2	3.590 (4)	H14···O1^v^	2.5700
C10···O1	3.154 (4)	H14···H8A	2.3500
C11···C15^iii^	3.592 (5)	H15B···C1	2.6400
C12···C15^iii^	3.399 (5)	H15B···C2	2.9700
C15···C1	3.303 (5)	H15B···C6	2.7500
C15···C11^iv^	3.592 (5)	H15C···O4	2.7500
C15···C12^iv^	3.399 (5)	H15C···C16	3.0500
C15···C6	3.572 (5)	H15C···H16C	2.5000
C16···O3^v^	3.336 (5)	H15C···C12^iv^	3.0100
C16···C3^iv^	3.574 (5)	H15C···C13^iv^	2.9500
C16···C4^iv^	3.423 (6)	H16A···O3^v^	2.4700
C1···H15B	2.6400	H16B···O2	2.8700
C2···H8A	2.8700	H16B···C8^vi^	2.9900
C2···H15B	2.9700	H16B···C9^vi^	2.7600
C3···H16C^iii^	2.8300	H16B···C10^vi^	2.9200
C4···H16C^iii^	2.7000	H16B···H8B^vi^	2.4900
C6···H15B	2.7500	H16C···H15C	2.5000
C8···H16B^vi^	2.9900	H16C···C3^iv^	2.8300
C8···H2	2.8900	H16C···C4^iv^	2.7000
			
O2—P1—O3	108.65 (15)	C4—C3—H3	120.00
O2—P1—O4	114.93 (14)	C3—C4—H4	120.00
O2—P1—C7	113.24 (14)	C5—C4—H4	120.00
O3—P1—O4	108.19 (15)	C4—C5—H5	120.00
O3—P1—C7	108.47 (14)	C6—C5—H5	120.00
O4—P1—C7	103.05 (13)	C1—C6—H6	120.00
P1—O3—C15	126.2 (3)	C5—C6—H6	120.00
P1—O4—C16	123.3 (2)	C7—C8—H8A	109.00
C7—O1—H1	109.00	C7—C8—H8B	109.00
C2—C1—C6	118.1 (3)	C9—C8—H8A	109.00
C6—C1—C7	120.3 (3)	C9—C8—H8B	109.00
C2—C1—C7	121.6 (3)	H8A—C8—H8B	108.00
C1—C2—C3	120.4 (3)	C9—C10—H10	119.00
C2—C3—C4	120.8 (3)	C11—C10—H10	120.00
C3—C4—C5	119.3 (3)	C10—C11—H11	120.00
C4—C5—C6	120.6 (4)	C12—C11—H11	120.00
C1—C6—C5	120.8 (3)	C11—C12—H12	120.00
P1—C7—C1	110.6 (2)	C13—C12—H12	120.00
P1—C7—C8	108.9 (2)	C12—C13—H13	120.00
O1—C7—C1	108.2 (2)	C14—C13—H13	120.00
O1—C7—C8	111.7 (2)	C9—C14—H14	119.00
C1—C7—C8	112.8 (2)	C13—C14—H14	119.00
P1—C7—O1	104.4 (2)	O3—C15—H15A	109.00
C7—C8—C9	114.9 (3)	O3—C15—H15B	109.00
C8—C9—C10	121.7 (3)	O3—C15—H15C	109.00
C10—C9—C14	117.4 (3)	H15A—C15—H15B	109.00
C8—C9—C14	120.9 (3)	H15A—C15—H15C	109.00
C9—C10—C11	121.0 (3)	H15B—C15—H15C	109.00
C10—C11—C12	120.2 (4)	O4—C16—H16A	110.00
C11—C12—C13	119.3 (4)	O4—C16—H16B	109.00
C12—C13—C14	120.7 (4)	O4—C16—H16C	110.00
C9—C14—C13	121.4 (4)	H16A—C16—H16B	109.00
C1—C2—H2	120.00	H16A—C16—H16C	110.00
C3—C2—H2	120.00	H16B—C16—H16C	109.00
C2—C3—H3	120.00		
			
O2—P1—O3—C15	158.6 (3)	C2—C1—C7—C8	−55.0 (4)
O4—P1—O3—C15	33.2 (3)	C6—C1—C7—P1	−110.6 (3)
C7—P1—O3—C15	−77.9 (3)	C6—C1—C7—O1	3.2 (4)
O2—P1—O4—C16	−47.0 (3)	C6—C1—C7—C8	127.3 (3)
O3—P1—O4—C16	74.6 (3)	C1—C2—C3—C4	−2.3 (5)
C7—P1—O4—C16	−170.7 (3)	C2—C3—C4—C5	−0.5 (6)
O2—P1—C7—O1	60.7 (2)	C3—C4—C5—C6	2.1 (6)
O2—P1—C7—C1	176.8 (2)	C4—C5—C6—C1	−0.9 (6)
O2—P1—C7—C8	−58.8 (2)	P1—C7—C8—C9	180.0 (2)
O3—P1—C7—O1	−60.0 (2)	O1—C7—C8—C9	65.2 (3)
O3—P1—C7—C1	56.1 (2)	C1—C7—C8—C9	−56.9 (3)
O3—P1—C7—C8	−179.5 (2)	C7—C8—C9—C10	−73.5 (4)
O4—P1—C7—O1	−174.51 (18)	C7—C8—C9—C14	109.9 (3)
O4—P1—C7—C1	−58.4 (2)	C8—C9—C10—C11	−177.6 (3)
O4—P1—C7—C8	66.0 (2)	C14—C9—C10—C11	−0.8 (5)
C6—C1—C2—C3	3.4 (4)	C8—C9—C14—C13	177.4 (3)
C7—C1—C2—C3	−174.4 (3)	C10—C9—C14—C13	0.6 (5)
C2—C1—C6—C5	−1.8 (5)	C9—C10—C11—C12	1.1 (5)
C7—C1—C6—C5	176.0 (3)	C10—C11—C12—C13	−1.2 (5)
C2—C1—C7—P1	67.1 (3)	C11—C12—C13—C14	1.0 (6)
C2—C1—C7—O1	−179.1 (3)	C12—C13—C14—C9	−0.7 (6)

Symmetry codes: (i) −*x*+2, −*y*, −*z*; (ii) *x*+1, *y*, *z*; (iii) *x*, −*y*+1/2, *z*+1/2; (iv) *x*, −*y*+1/2, *z*−1/2; (v) *x*−1, *y*, *z*; (vi) −*x*+1, −*y*, −*z*; (vii) −*x*+2, *y*−1/2, −*z*+1/2; (viii) −*x*+2, *y*+1/2, −*z*+1/2.

### Hydrogen-bond geometry (Å, °)

**Table:**

*D*—H···*A*	*D*—H	H···*A*	*D*···*A*	*D*—H···*A*
O1—H1···O2^i^	0.8200	1.9000	2.710 (3)	172.00
C2—H2···O4	0.9300	2.5200	2.956 (4)	109.00
C6—H6···O1	0.9300	2.3000	2.671 (4)	103.00
C14—H14···O1^v^	0.9300	2.5700	3.432 (5)	154.00
C16—H16A···O3^v^	0.9600	2.4700	3.336 (5)	149.00
C15—H15B···CgA	0.9600	2.7200	3.608 (5)	154.00

Symmetry codes: (i) −*x*+2, −*y*, −*z*; (v) *x*−1, *y*, *z*.

## References

[bb1] Enraf–Nonius (1994). *CAD-4 EXPRESS* Enraf–Nonius, Delft, The Netherlands.

[bb2] Farrugia, L. J. (1997). *J. Appl. Cryst.* **30**, 565.

[bb3] Farrugia, L. J. (1999). *J. Appl. Cryst.* **32**, 837–838.

[bb4] Harms, K. & Wocadlo, S. (1995). *XCAD4* University of Marburg, Germany.

[bb5] Hudson, H. R., McPartlin, M., Matthews, R. W., Powell, H. R., Yusuf, R. O., Jaszay, Z. M., Keglevich, G., Petnehazy, I. & Toke, L. (1993). *Phosphorus Sulfur Silicon Relat. Elem* **79**, 239–243.

[bb6] North, A. C. T., Phillips, D. C. & Mathews, F. S. (1968). *Acta Cryst.* A**24**, 351–359.

[bb7] Sheldrick, G. M. (2008). *Acta Cryst.* A**64**, 112–122. 10.1107/S0108767307043930 18156677

[bb8] Spek, A. L. (2003). *J. Appl. Cryst.* **36**, 7–13.

[bb9] Tahir, M. N., Acar, N., Yilmaz, H., Danish, M. & Ülkü, D. (2007). *Acta Cryst.* E**63**, o3817–o3818.

[bb10] Wroblewski, A. E., Maniukiewicz, W. & Karolczak, W. (2000). *J. Chem. Soc. Perkin Trans. 1*, pp. 1433–1435.

